# Brain type carnosinase in dementia: a pilot study

**DOI:** 10.1186/1471-2377-7-38

**Published:** 2007-11-05

**Authors:** Cynthia M Balion, Carolyn Benson, Parminder S Raina, Alexandra Papaioannou, Christopher Patterson, Afisi S Ismaila

**Affiliations:** 1 Department of Laboratory Medicine, Hamilton Health Sciences, Hamilton, Ontario, Canada; 2 Department of Pathology and Molecular Medicine, McMaster University, Hamilton, Ontario, Canada; 3 Department of Medicine, McMaster University, Hamilton, Ontario, Canada; 4 Department of Clinical Epidemiology & Biostatistics, McMaster University, Hamilton, Ontario, Canada

## Abstract

**Background:**

The pathological processes underlying dementia are poorly understood and so are the markers which identify them. Carnosinase is a dipeptidase found almost exclusively in brain and serum. Carnosinase and its substrate carnosine have been linked to neuropathophysiological processes.

**Methods:**

Carnosinase activity was measured by a flourometric method in 37 patients attending a Geriatric Outpatient Clinic. There were 17 patients without dementia, 13 had Alzheimer's disease (AD) and 7 had mixed dementia (MD).

**Results:**

The range of serum carnosinase activity for patients without dementia was 14.5 – 78.5 μmol/ml/h. There was no difference in carnosinase activity between patients without dementia (40.3 ± 15.2 μmol/ml/h) and patients with AD (44.4 ± 12.4 μmol/ml/h) or MD (26.6 ± 15 μmol/ml/h). However, levels in the MD group were significantly lower than the AD group (p = 0.01). This difference remained significant after adjusting for gender, MMSE score, exercise, but not age, one at a time and all combined. The effect of other medical conditions did not remove the significance between the AD and MD groups. The MD group, but not the AD group, demonstrated a significant trend with carnosinase activity decreasing with duration of disease (from first recorded date of diagnosis to date of blood collection) (r = -0.76, p = 0.049). There was no association with carnosinase activity and MMSE score in the AD or MD group. Both AD and MD patients on any dementia medication (donepezil, galantamine, memantine) had higher carnosinase activity compared to those not taking a dementia medication. Carnosinase activity was higher in patients who regularly exercised (n = 20) compared to those who did not exercise regularly (n = 17)(p = 0.006).

**Conclusion:**

This exploratory study has shown altered activities of the enzyme carnosinase in patients with dementia.

## Background

Dementia has emerged as a major clinical, societal and economic problem, especially in industrialized societies. Alzheimer's disease (AD) is the most common form of dementia followed by vascular dementia and mixed dementia (AD with cerebrovascular disease). Early and accurate diagnosis is desirable, as current therapies are most effective in the early stages [1]. Early diagnosis also allows the cognitively aware patient to deal with future issues in medical care, safety, and legal matters [2].

Numerous plasma and serum markers for dementia have been identified, and many show significant differences between patients with dementia and those without dementia, but they lack the sensitivity and specificity needed for diagnosis. Furthermore, no biomarker has yet demonstrated the utility to monitor the progress of the disease, response to therapy or to predict outcome. Most of the proposed biomarkers are not brain specific but reflect other pathologies involving coagulation and inflammation [3], immunological [4], detoxification [5], oxidative stress [6], lipid metabolism and vascular disease [6,7]. Although proteins specific to the neuropathology of amyloid plaques such as amyloid β-protein (Aβ) and amyloid precursor protein (APP) are detectable in peripheral blood, they have shown little value in diagnosis or monitoring therapy [6].

Successful markers of dementia will most likely be linked to pathological processes within affected brain cells. Carnosinase (CN1; EC 3.4.13.20) is an enzyme found almost exclusively in brain tissue and serum [8]. It is a member of the M20 metalloproteinase family and specifically degrades dipeptides. Two of these, carnosine (β-alanyl-histidine) and homocarnosine (γ-aminobutyryl-histidine), have been linked to aging and dementia.

Carnosine has many reported functions including intracellular buffer, metal- chelator (particularly copper), anti-oxidant, and free radical scavenger [9]. It reacts with protein carbonyl groups serving as an alternative to glucose, reversing the glycation process at the Schiff base stage [10]. Carnosine, through its copper chelating and anti-glycating function, inhibits the production of advanced glycation endproducts (AGEs) [11]. AGEs result from the reaction of amino groups with reducing sugars resulting in irreversibly cross-link proteins. While AGEs are considered part of the normal aging process [12], accelerated production is implicated in the formation of β-amyloid plaques and neurofibrillary tangles [11]. Carnosine may also serve as an anti-senescence agent since it has been shown to extend the life of human diploid fibroblasts [13] and reduce the rate for telomeric shortening and damage to DNA of human fetal lung fibroblasts [14].

In the olfactory bulb, carnosine prevents the inhibitory effects of copper on synaptic transmission through chelation, thus acting as a neuromodulator [15]. Since AD is characterized by early changes in the olfactory system [16] and high levels of copper are associated with senile plaques and neurofibrillary tangles [17], lower levels of carnosine could decrease copper chelation and impair olfaction through decreased synaptic transmission [15] and neurotoxic effects [18].

Homocarnosine, a specific substrate of carnosinase is degraded into γ-aminobutyric acid (GABA) and L-histidine. Post-mortem and imaging studies of AD have shown decreases in GABA in the temporal, frontal, and parietal regions [19]. Decreased histidine levels in the frontal and temporal lobes of AD patients have also been reported [20]. Since histidine is produced when carnosine and homocarnosine is cleaved by carnosinase, this observation may reflect decreased carnosinase activity.

Case studies of carnosinase deficiency have reported symptoms that include progressive mental deficiency, non-progressive mental retardation, developmental delay, spastic paraplegia, seizures, neurosensory hearing loss, retinitis pigmentosa, and progressive childhood dementia [21,22]. Reduced activity levels have been found in other neurological disorders including Parkinson's disease, multiple sclerosis, and following a cerebrovascular event [23,24]. Recently, serum carnosinase levels have been shown to decrease during cardiopulmonary bypass surgery [25]. Ischemia is a major complication of cardiac surgery and a decrease in carnosinase during this procedure may be a neuroprotective mechanism [25] as carnosine may reduce neurotoxicity through its antioxidant capacity [18]. Brains afflicted with AD show biochemical evidence of ischemia, specifically Aβ deposits and an increase in basic fibroblastic growth factor [26]. Decreased blood flow and oxygenation has been documented in the temporal lobes in AD [27]. These vascular changes may initiate the same decrease in carnosinase activity as seen in patients with cerebrovascular events. Carnosine has also been shown to produce vascular relaxation whereas its constituent amino acid β-alanine produced vasoconstriction [28].

In light of this growing body of information relating carnosinase and its substrates to dementia, we undertook an exploratory study to investigate carnosinase activity in patients with dementia.

## Methods

### Participants

After approval by the Hamilton Health Sciences/McMaster Faculty of Health Sciences Research Ethics Board, we studied 37 patients who attended a Geriatric Outpatient Clinic at Hamilton Health Sciences between July 2004 and April 2005. Informed consent was obtained from the patient or relative. A questionnaire was administered to patients collecting data about their family history of disorders, diet, exercise frequency, and recent weight loss. All prescriptions, over-the-counter medications and supplements were recorded. Medical history was obtained and confirmed through medical records. The most recent mini-mental state examination (MMSE) score was used as a global measure of cognition [29]. The diagnosis of dementia was based upon the NINCDS-ADRDA criteria [30] incorporating clinical assessment, laboratory and neuroimaging studies where indicated.

### Carnosinase activity

Blood samples were collected by venipuncture. Serum was separated after samples were centrifuged at 2,000 × *g *for 10 min at room temperature, aliquoted and stored at -20°C until analysis. Serum carnosinase activity was assayed by the method described by Lenney et al. [31]. All reagents were purchased from Sigma-Aldrich Canada Ltd. (Oakville, ON). Serum (50 μl) was first incubated at 30°C with 250 μl NH_4_OH (pH 8.5) and 150 μl CdCl for 10 min. The enzyme reaction was started by adding 100 μl of 100 mM carnosine and allowed to proceed for 30 min at 30°C. At this point the reaction was stopped by adding 500 μl of 0.6 M TCA. The solution was centrifuged 800 × *g *for 10 min and the supernatant removed. L-Histidine was measured fluorometrically by the method described by Ambrose et al. [32]. Briefly, 50 μl of supernatant was combined with 1.2 ml 0.83 N NaOH and 500 μl 0.2% *o*-phthalaldehyde (OPA) and incubated at 30°C for 15 min. The reaction was stopped by adding 500 μl 4.0 M H_3_PO_4 _and incubated for a further 15 min at 30°C. The solution was cooled at room temperature for 1 hour and read on a SpectraMax M2 spectrophotofluorometer (Molecular Devices, Sunnyvale, CA) at an excitation wavelength of 340 nm and emission wavelength of 450 nm.

### Standard curves and detection limits

Standard curves were created using L-histidine and water in place of TCA supernatant in the *o*-phthalaldehyde assay. Standard curves using combinations of carnosine, β-alanine, and L-histidine representing different levels of hydrolysis were not significantly different from standard curves with L-histidine and water [33]. Carnosinase activity was calculated using the L-histidine standard curve and was expressed as μmol of L-histidine produced per ml of serum per hour. The lowest limit of detection was determined by adding 3 SD to the mean fluorescence of 20 blank replicates, which corresponded to an enzyme activity of 4 μmol/ml/h.

### Statistical analysis

Normal distribution of the data was done using the Kolmogorov-Smirnov test and Shapiro-Wilk tests. The Pearson correlation was calculated for relationships among serum carnosinase activity, duration of dementia and MMSE score. The software used for these analyses was Analyze-it for Microsoft Excel (Analyze-it Software, Ltd., Leeds, England). Group comparisons were performed using the student's t-test, regression analysis and analysis of variance (SPSS 12.0, SPSS Inc., Chicago, IL). Comparisons were considered to be significant when p < 0.05.

## Results

The study population consisted of 17 patients without dementia and 20 patients with dementia. The dementia group included 13 patients with AD and 7 patients with MD. There were more females (n = 29) than males (n = 8) in all groups. There were no significant differences in age between any groups (p > 0.05). Demographic and clinical characteristic data are presented in Table 1.

**Table 1:** Demographic and clinical characteristics of the without dementia, AD and MD groups. AD, Alzheimer's disease; MD, mixed dementia

	**Patient group**		
	**Without dementia**	**AD**	**MD**

Number of patients	17	13	7
Mean age (range)	80.2 (64–92)	75.5 (58–88)	82.3(72–92)
Female gender	15	8	6
Mean aterial pressure (mmHg)	99 ± 13	103 ± 9	97 ± 7
Duration of dementia (month)	-	19 ± 18	11 ± 10
Mini-mental state examination (MMSE) score	-	20 ± 7	21 ± 7
Neuroimaging (CT or MRI)	2	5	4
Diet, meat eaten within the last day	13	11	4
Exercise, 3 or more times per week	9	9	2
Medical Conditions:			
Arthritis	8	4	4
Cardiovascular disease	5	4	3
Depression	4	2	2
Diabetes	3	1	1
Hypothyroidism, treated	2	1	0
Parkinson's disease	1	0	0
Renal insufficiency	0	0	2
Stroke, history	2	0	0
TIA, history	0	1	2
Dementia medication:			
Donepezil	0	6	2
Memantine	0	1	0
Galantamine	0	1	2

AD, Alzheimer's disease; MD, mixed dementia.

**Figure 1:**
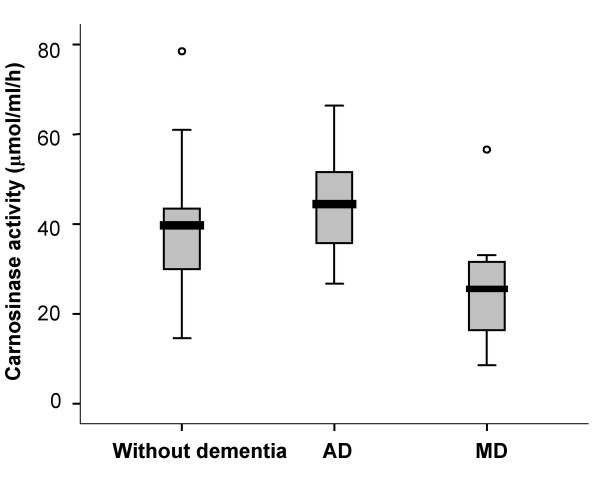
**Box-and-whisker plot showing carnosinase activity in without dementia (n = 17), AD (n = 13), and MD (n = 7) groups**. Comparisons were not significant between without dementia and AD or MD groups, however the difference between the AD and MD groups was significant (p = 0.01).

Carnosinase activity for patients without dementia was 14.5 – 78.5 μmol/ml/h, comparable to the adult range determined by Lenney et al. (18 – 72 μmol/ml/h) using the same method [31]. The results of the analysis of variance showed a significant difference in means across groups (p = 0.04). Further analysis showed there was no difference in carnosinase activity between patients without dementia (40.3 ± 15.2 μmol/ml/h) and patients with AD (44.4 ± 12.4 μmol/ml/h) or MD (26.6 ± 15 μmol/ml/h) (Figure 1). However, the difference between the AD and MD groups was significant (mean difference = 17.8 μmol/ml/h, p = 0.01).

Table 2 shows the pairwise comparisons after adjusting for age, gender, MMSE score and exercise (one variable at a time and all variables combined). The results show there was a significant difference in the means of carnosinase between AD and MD groups after adjusting for each variable except for age (p = 0.06). The effect of medical conditions, other than dementia on carnosinase activity, was tested by comparison of groups (unpaired t-test) after removal of each medical condition listed in Table 1. Table 3 shows that the AD group remains significantly different (p < 0.05) from the MD group regardless of which medical condition is removed.

**Table 2:** Pairwise comparisons between without dementia, AD and MD groups. These comparisons have been adjusted for age, gender, exercise, MMSE score and all four variables combined. Carnosinase activity is expressed as mean difference (SE)

**Variable**	**Carnosinase (μmol/ml/h)**		
	**Without dementia minus MD**	**Without dementia minus AD**	**AD minus MD**

None	12.3 (6.4)	-5.5 (5.2)	17.8 (6.7)**
Age	10.8 (6.0)	-2.1 (5.1)	12.8 (6.5)‡
Gender	12.2 (6.4)	-6.3 (5.5)	18.6 (6.9)**
Exercise	9.8 (6.1)	-3.7 (5.0)	13.5 (6.5)*
MMSE score	1.7 (7.6)†	-16.4 (7.2)	18.1 (5.9)**
Combined	-1.9 (6.9)	-14.7 (7.2)	12.8 (6.1)*

AD, Alzheimer's disease; MD, mixed dementia.

* p-value < 0.05, ** p-value = 0.01, † p-value = 0.06 between AD and MD

‡ p-value < 0.05 between MD and without dementia

**Table 3:** Mean carnosinase activity in the without dementia, AD and MD groups after removal of medical conditions (Table 1)

**Medical condition removed**	**Carnosinase (μmol/ml/h)**		
	**Without dementia**	**AD**	**MD**

None	40.3	44.4	26.6**
Arthritis	35.8	47.0	22.0*
Cardiovascular disease	41.2	41.6	21.4*
Depression	41.5	44.5	26.2*
Diabetes	36.6	44.5	28.7*
Hypothyroidism, treated	40.3	42.6	26.6*
Parkinson's Disease	38.9	44.4	26.6**
Renal insufficiency	39.0	44.4	27.6*
Stroke, history	40.3	44.4	26.6**
TIA, history	39.0	43.2	19.4‡***

AD, Alzheimer's disease; MD, mixed dementia.

* p-value < 0.05, ** p-value = 0.01, *** p-value = 0.001 between AD and MD

‡ p-value = 0.01 between MD and without dementia

**Figure 2:**
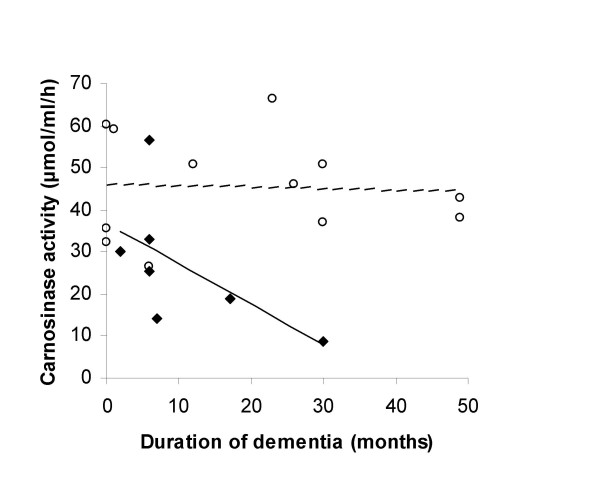
**Relationship between the duration of dementia and carnosinase activity in patients with MD (◆, n = 7) and AD (○, n = 12)**. Duration is the time from the first recorded date of diagnosis to the date of blood collection. Duration of dementia was significant for the MD group (--, r = -0.76, p = 0.05) but not for the AD group (----, r = -0.06, p = 0.86).

**Figure 3:**
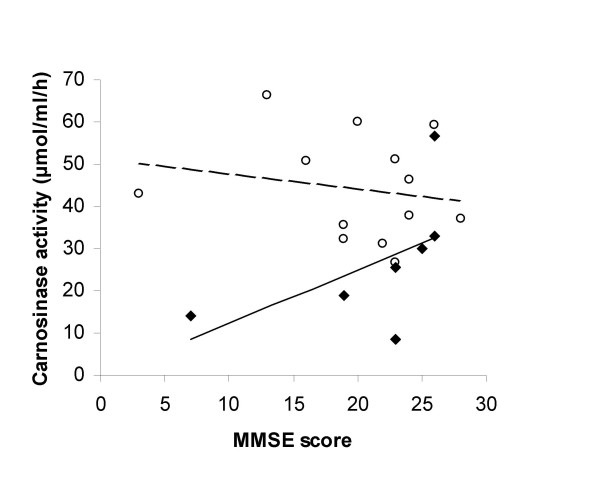
**Relationship between MMSE score and carnosinase activity with MD (◆, n = 7) and AD (○, n = 13)**. MMSE score were not related to carnosinase activity for the MD (--, r = 0.55, p = 0.21) or the AD group (----, r = -0.19, p = 0.54).

**Figure 4:**
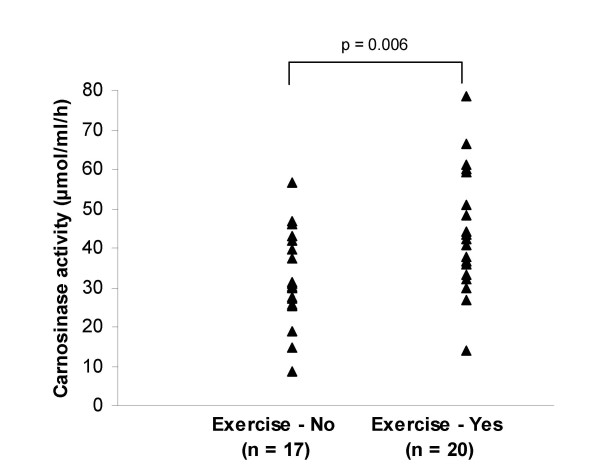
**Carnosinase activity in patients who exercised regularly (three or more times per week) compared to those who did not exercise regularly**. The mean carnosinase activity (μmol/ml/h) and 95% confidence intervals for the groups were: No = 31.4 (25.3 to 37.5), Yes = 44.6 (37.5 to 51.7).

Further analysis of the MD group demonstrated a significant trend with carnosinase activity decreasing with duration of disease (from first recorded date of diagnosis to date of blood collection) (r = -0.76, p = 0.05) (Figure 2). There was a positive association with carnosinase activity and MMSE scores, although not significant (r = 0.55, p = 0.21) (Figure 3). There was no significant association in the AD group between carnosinase activity and duration of disease (r = -0.06, p = 0.86) or with MMSE score (r = -0.19, p = 0.54). Diet containing meat, a source of carnosine, or use of dementia medications (donepezil, galantamine, memantine) was not associated with a difference in carnosinase activity between patients with dementia (AD and MD) and those without dementia (p > 0.05). However, carnosinase values were higher in both AD and MD for the patients on dementia medications compared to those who were not on dementia medications. Significance between groups was reached for AD (p = 0.05), but not for MD (p = 0.59). Diet containing meat did not show a difference within the AD or MD groups. Exercise on the day of blood collection showed no difference between patients with dementia and those without dementia, but exercising three or more times a week was associated with an increase in carnosinase activity (n = 37, p = 0.006) (Figure 4).

## Discussion

In this study we have shown that levels of carnosinase activity have the potential to differentiate AD from MD with very little overlap between these conditions. As the only known difference between these groups was the presence or absence of cerebrovascular disease, this suggests that vascular pathology may be the reason for lower carnosinase activity. Vascular dementia is associated with cerebrovascular accident, a condition known to lower carnosinase activity [23,24]. Furthermore, compared to patients without dementia, carnosinase activity remains significantly decreased three months after a cerebrovascular accident [23]. Decreased levels have also been found in Parkinson's disease [24]. In an autopsy series of over 700 Parkinsonism patients, 19% had co-existing cerebrovascular lesions, and 20% of secondary Parkinsonism patients had vascular lesions [34]. Our current findings allow speculation that carnosinase could differentiate "pure" AD from vascular dementia, which cannot be achieved with either CSF Aβ1–42 or CSF total tau [1]. Further evidence supporting a vascular role for carnosinase comes from a recent study showing that its substrate carnosine causes vascular relaxation independent of the endothelium [28].

Several reasons have been suggested for decreased carnosinase levels in neurological disorders. Wassif et al. [24] suggested that a disruption of the blood brain barrier (BBB) could result in reduced activity of carnosinase seen in several disorders of the CNS. This idea is supported by the ability of cerebral ischemia to alter the BBB, specifically changing endothelial permeability and basal lamina integrity [35]. The association of multiple sclerosis with BBB hyperpermeability further supports this idea [36,37]. Skoog [37] found higher CSF/serum albumin ratios in several types of dementia including AD and vascular dementia indicating an impairment of the BBB. Recently, aggregation of Aβ has been associated with damage to the structural microvascular and BBB abnormalities in an AD mouse model [38]. Damage to carnosinase-producing cells could be another reason for reduced carnosinase activity. In stroke patients, Butterworth et al. [23] found no relationship between size of the infarct and carnosinase activity. If death of carnosinase producing cells were responsible one would expect more cell death to cause a greater decrease in carnosinase activity.

Schoen [25] suggested that the decreased levels of carnosinase seen during cardiopulmonary bypass surgery were protective as both carnosine and homocarnosine can protect neurons against ischemia and oxidative stress. On the other hand, carnosinase degrades homocarnosine to GABA, an inhibitory neurotransmitter. Lower GABA levels may lead to increased neuronal damage and death. In one study of patients who had suffered a stroke, lower carnosinase activity was correlated with poorer outcome [23].

A genetic basis for lower carnosinase activity is also possible. The gene for serum carnosinase is located on 18q22.3 [8]. A child with serum carnosinase deficiency was found to have a deletion distal to 18q21.3 [39] and recently a locus on chromosome 18 was identified for familial AD in Caribbean Hispanics [40]. These two reports, along with a study showing an association between the allelic variation of this gene and carnosinase activity [41], lead to the speculation that lower carnosinase activity could be genetic. In our study, patients with MD may have always had low carnosinase activity and thus susceptible to dementia. Down-regulation of carnosinase mRNA is an alternative hypothesis for lower carnosinase levels. Gene regulation has been shown for the AD marker neural thread protein (AD7C-NTP). It is up regulated in the brains and CSF of patients with AD compared to controls [42].

In our study, carnosinase activity in MD was related to the duration of dementia. This raises the possibility that carnosinase may have a role in monitoring the progression and therapy of patients with MD. Furthermore, patients treated with dementia medications had somewhat higher carnosinase activities in both AD and MD groups (data not shown). Regular exercise was also associated with increased carnosinase activity and regular physical activity has been associated with a decreased risk of vascular dementia in women [43].

In contrast to other biomarkers for dementia, which are measured in CSF, carnosinase is measured in the serum, which is advantageous as venipuncture is far less invasive than lumbar puncture. Carnosinase activity in CSF is about 30-fold higher than the normal serum/CSF protein ratio indicative of a predominantly brain-derived source of carnosinase in CSF [24]. Although studies have not been done to determine the source of serum carnosinase, carnosinase (CN1) is almost exclusively localized to brain tissue.

It is important to comment on the method chosen to measure carnosinase, as there are several methods reported in the literature, making comparisons between studies difficult. Optimization studies for measuring carnosinase activity were previously performed [33] showing that maximum activity occurs at a pH of 8.5 with Cd^2+ ^as the metal ion cofactor and confirms those of Lenney et al. [31]. Although Mn^2+ ^is often used as the cofactor carnosinase activity is about 4-fold lower compared to Cd^2+^. Carnosine, although not a specific substrate for carnosinase, is preferred over homocarnosine as it is hydrolyzed more rapidly. These conditions essentially prevent any activity from the related cytosolic nonspecific dipeptidase (previously known as tissue carnosinase) from occurring [44]. Therefore, this assay method achieves high analytical specificity and sensitivity allowing small changes in activity to be detected. These characteristics are beneficial not only for diagnosis but also for monitoring changes in the disease state and treatment.

Since this study was exploratory in nature, designed to see if serum carnosinase activity was altered in dementia, the sample size was small and limited the use of multiple regression analysis to adjust for potential confounding variables. The potential for age, gender, medications and other comorbidities to affect carnosinase activity cannot be ruled out. However, apart from age, univariate analyses for gender, MMSE score and exercise did not change the results. There is limited data in the literature on the effect of age or gender on carnosinase activity [31,33,45,46]. In general, carnosinase activity is absent or low in newborns and gradually increases until adulthood. Only two small studies examined gender and found no difference [31,33]. This study, the first to report carnosinase activity in elderly adults, did not show a difference in activity compared to younger adult populations [31]. However, a larger group of elderly persons is required to show whether there may be an age or gender association with carnosinase activity.

In this study, there were a few patients with medical conditions that have been associated with lower values of carnosinase activity (e.g., stroke, Parkinson's disease). No patients in the AD and MD groups had any of these conditions. The number of other medical conditions in any group was too few to adjust for using regression analysis, however, the AD group remained significantly different from the MD group if any one of these exclusions was made.

It is also possible that in the absence of post-mortem confirmation of diagnosis, some patients may have been misclassified. Diagnosis was made by mostly clinical assessment and only nine of the twenty patients with dementia had neuroimaging. Neuroimaging does not necessarily improve diagnostic accuracy nor is it required by published criteria for the diagnosis of dementia or AD (though frequently included in the diagnostic evaluation) [47]. For example, in a study of 501 patients evaluated for dementia, 375 had a CT scan. In these 375 patients, 28% of the diagnoses during life were incorrect when compared with post mortem pathology. In those who did not undergo CT scanning the diagnosis during life was incorrect only 18% of the time [48], Furthermore, CT scans may both overestimate the presence of strokes [49] and fail to identify them [50].

Carnosinase activity appears to play a role in dementia. Lower carnosinase activity could be a biochemical response for neuroprotection, a genetic variation in the enzyme, or both. Further research is needed to determine whether carnosinase fulfils the potential for a diagnostic and disease-monitoring measure for dementia.

## Abbreviations

Aβ, amyloid β-protein; AD, Alzheimer's disease; AGEs, advanced glycation endproducts; APP, amyloid precursor protein; GABA, γ-aminobutyric acid; MD, mixed dementia; MMSE, mini-mental state examination; OPA, *o*-phthalaldehyde.

## Competing interests

The author(s) declare that they have no competing interests.

## Authors' contributions

CMB contributed to the conception, design, laboratory analyses, analysis of data, drafting, writing and revising of the article. CB contributed to laboratory analyses, and writing of the article. PSR contributed to the conception, design, analysis of data, drafting and revising of the article. AP contributed to patient recruitment and writing and revising of the article. CP contributed to patient recruitment and writing and revising of the article. ASI contributed to analysis of data and writing and revising of the article.

## Pre-publication history

The pre-publication history for this paper can be accessed here:


